# COVID-19 inactivated booster vaccines elicit strong protection against SARS-CoV-2 wild-type and Omicron variant in patients with breast cancer

**DOI:** 10.3389/fmed.2025.1516492

**Published:** 2025-04-01

**Authors:** Xiaomeng Li, Yali Xu, Haolong Li, Linrong Li, Yongmei Liu, Haoting Zhan, Qiang Sun, Yongzhe Li

**Affiliations:** ^1^Department of Clinical Laboratory, State Key Laboratory of Complex Severe and Rare Diseases, Peking Union Medical College Hospital, Chinese Academy of Medical Science and Peking Union Medical College, Beijing, China; ^2^Department of Clinical Laboratory, Peking University People's Hospital, Beijing, China; ^3^Department of Breast Surgery, Peking Union Medical College Hospital, Chinese Academy of Medical Science and Peking Union Medical College, Beijing, China

**Keywords:** breast cancer, SARS-CoV-2, antibody, vaccination, immune response

## Abstract

**Background:**

Patients with breast cancer are at an increased risk of severe COVID-19 and related mortality. However, the ability of inactivated vaccine-induced antibodies to neutralize both SARS-CoV-2 and its Omicron variant following a third SARS-CoV-2 vaccine dose remains unclear in these patients.

**Methods:**

Blood samples from 211 breast cancer patients and 155 healthy controls were analyzed after one, two, or three doses of the inactivated SARS-CoV-2 vaccine. Levels of total anti-SARS-CoV-2 antibodies, anti-receptor binding domain (RBD) IgG, and neutralizing antibodies (NAbs) against both the SARS-CoV-2 wild-type virus and the BA.4/BA.5 (Omicron) variant, along with lymphocyte subsets, were measured 2 weeks to 3 months and more than 6 months after the second and third vaccinations, respectively.

**Results:**

Levels of anti-RBD IgG and NAb inhibition rates against both the SARS-CoV-2 wild-type virus and the BA.4/BA.5 (Omicron) variant were significantly higher in breast cancer patients after the third dose than after the second dose. However, these levels remained lower than those observed in healthy controls. Univariate analysis revealed that >6 months after receiving two or three doses was associated with undetectable NAbs against the SARS-CoV-2 wild-type virus compared to those at 2 weeks to 3 months. Additionally, patients aged 60 years or older were correlated with undetectable NAbs against the BA.4/BA.5 (Omicron) variant. Immune responses after two or three doses were not affected by endocrine therapy, either current therapy or vaccination. In particular, univariate and multivariate analyses revealed that the vaccination of breast cancer patients with CoronaVac resulted in significantly higher NAb inhibition rates against the SARS-CoV-2 wild-type virus than BBIBP-CorV.

**Conclusion:**

Breast cancer patients boosted with a third dose of inactivated vaccines demonstrated the potent neutralization of SARS-CoV-2 and the Omicron variant compared to receiving one or two doses. Vaccination-mediated NAb induction was affected by age, time > 6 months after vaccination, vaccine type, and cancer-targeted treatment. Therefore, the study results indicated an urgent need for caution and additional strategies to protect these patients.

## Introduction

Cancer patients are vulnerable to SARS-CoV-2. Although anti-SARS-CoV-2 spike receptor-binding domain (RBD) IgG antibody titers in cancer patients undergoing treatment have been reported to be significantly lower than those in healthy controls, their antibody responses to the COVID-19 vaccine remain significant (1). Full vaccination or booster vaccine doses have been shown to protect cancer patients against morbidity and mortality from COVID-19 compared to no vaccination during the Omicron phase (2). Patients with solid cancer who received a booster dose of the vaccine demonstrated higher virus-neutralizing capacity against the Omicron variant than those receiving only two doses (3).

Breast cancer is the most commonly diagnosed cancer worldwide (4). The immunogenicity of SARS-CoV-2 vaccines in this disease has caused considerable concern. The majority of patients with breast cancer exhibit adequate anti-spike and anti-RBD IgG antibodies after two or three SARS-CoV-2 vaccinations, although their antibody responses were lower than in non-cancer controls (5–9). Neutralizing antibody levels are high with immune protection. After the first dose, SARS-CoV-2 neutralizing antibody (NAb) levels in patients with breast cancer were similar to those in healthy controls (10). In particular, Omicron variant neutralization was low even after the second vaccination (5). Chemotherapy and targeted therapy for breast cancer patients affect the antibody response level (5, 7, 9). However, the induction of SARS-CoV-2 NAbs after a booster dose in breast cancer patients, particularly their immunity against the Omicron variant, and clinical features affecting the induction of these antibodies remain unclear. Less is known about how boosters elicit more immune protection in these patients than healthy controls. Therefore, understanding the determinants affecting the vaccine-mediated induction of NAbs in breast cancer patients is critical for developing effective measures against the SARS-CoV-2 virus.

The current study assessed the association of inactivated vaccines with anti-SARS-CoV-2 total antibodies, anti-RBD IgG antibodies, and NAbs against the SARS-CoV-2 wild-type virus and the BA.4/BA.5 (Omicron) variant. Then, inactivated vaccine efficacy over time in breast cancer patients was compared against healthy controls after two and three vaccine doses.

## Materials and methods

### Study design and participants

The current cross-sectional and longitudinal cohort study included female breast cancer patients, patients with a history of confirmed breast cancer, age over 18 years, with no previous COVID-19 infection, who received the inactivated SARS-CoV-2 vaccine (CoronaVac or BBIBP-CorV). Healthy controls had no history of cancer, known inflammatory diseases, or any relevant medical conditions. Blood samples were collected in EDTA tubes at five time points: (1) after dose1 vaccination, (2) 2 weeks to 3 months after dose 2 vaccination (peak response), (3) > 6 months after dose 2 vaccination; (4) 2 weeks to 3 months after dose 3 vaccination (peak response), and (5) > 6 months after dose 3 vaccination. The clinical data were retrieved from the hospital information system. The clinical TNM stage, histology, histological grade, and molecular subtype were determined according to the Chinese Society of Clinical Oncology and National Comprehensive Cancer Network guidelines by referring to the pathological reports of surgical specimens. The study was approved by the Ethics Committee of Peking Union Medical College Hospital (K1524-K22C0665).

### Immunological assays

Plasma samples were used to evaluate humoral responses. Anti-SARS-CoV-2 total antibodies were assessed using ELISA kits from Beijing Wantai BioPharm (ws-1096, Beijing, China). An OD value of 0.19 was the positive cutoff value. Anti-SARS-CoV-2 total antibodies were quantified using the OD value of the samples/OD value of the cutoff (S/CO), and S/CO ≥ 1.00 was considered positive. ELISA kits manufactured by Hangzhou Proprium Biotech (05030001, Hangzhou, China) were used to quantify and measure SARS-CoV-2 anti-RBD IgG levels. A level of 11.6 BAU/mL was considered positive. The NAb assay was based on surrogate assays for competitive binding between RBD and its cellular receptor angiotensin-converting enzyme-2 (ACE2). NAbs were measured against the SARS-CoV-2 wild-type virus and the BA.4/BA.5 (Omicron) variant using the FDA-approved cPass™ SARS CoV-2 NAbs Detection ELISA Kit (L00847-A, GenScript, Piscataway, NJ, USA) as previously described (11). Inhibition rates ≥30% were considered to detect SARS-CoV-2 NAb.

Lymphocyte subsets (CD3^+^CD4^+^T cells, CD3^+^CD8^+^T cells, CD19^+^B cells, and CD16^+^CD56^+^NK cells) were assayed in fresh whole blood samples using flow cytometry (Beckman Coulter, USA) with specific monoclonal antibodies (CD3-FITC, CD (16 + 56)-PE, CD45-FITC, CD4-PE, CD8-ECD, CD3-PC5, CD3-APC-A750, CD45-FITC, and CD19-PE; A07735, 6607013, C41176, C41137, A07769, Beckman Coulter, USA). Detailed information on gating strategies and representative flow plots is provided in Supplementary Figure S3.

### Statistical analysis

Continuous data were analyzed and compared using the unpaired, two-tailed *t*-test for normally distributed data or the Mann–Whitney *U*-test for non-normally distributed data between the two groups. One-way analysis of variance (ANOVA) followed by Tukey’s multiple comparisons test (normally distributed data) or the Kruskal–Wallis test followed by Dunn’s multiple comparisons test (non-normally distributed data) were performed and compared among the various groups. Categorical variables were compared using the chi-squared test or Fisher’s exact test. Spearman’s rank correlation test helped perform correlations between the assays. Binomial logistic regression was utilized to examine the factors associated with positive NAb and anti-RBD IgG responses. In a multivariate regression model, a *p-*value of <0.1 led to inclusion in the univariate analysis. The analyses were performed using GraphPad Prism 9 (GraphPad Software, La Jolla, CA, USA) and SPSS Statistics 26 (IBM, Chicago, IL, USA) software. The data were considered statistically significant at *p* < 0.05.

## Results

### Characteristics of breast cancer patients and healthy controls

Healthy controls (*n* = 155) and breast cancer patients (*n* = 211) were recruited from the Peking Union Medical College Hospital between 10 June 2021 and 12 September 2022. The characteristics of the individuals are represented in Supplementary Table S1. The vaccinated breast cancer patients were older than the healthy vaccinated controls. More individuals received the same CoronaVac inactivated vaccine than those who received the same BBIBP-CorV inactivated vaccine. In contrast, a small proportion received both the CoronaVac and BBIBP-CorV inactivated vaccines. No statistically significant differences were observed in the inactivated vaccine type or the mean or median time to collect blood samples following matched vaccination. In the cross-sectional cohort study, blood samples were collected at five time points, and they were not matched with samples to investigate how inactivated vaccine efficacy changed over time in breast cancer patients. Blood samples were collected only at four time points for healthy controls and were not matched. The clinical characteristics of the patients are presented in Supplementary Table S1. In the longitudinal cohort study, matched samples were available in five cases among the breast cancer patients after the first, second, or third vaccine doses. The blood samples of one patient (patient 1) were collected at three different time points: (1) after dose 1 vaccination, (2) > 6 months after dose 2 vaccination, and (3) > 6 months after dose 3 vaccination. The blood samples of two patients (patients 2 and 4) were both collected at two different time points: (1) 2 weeks to 3 months after dose 2 vaccination and (2) > 6 months after dose 2 vaccination. The blood samples of the third patient (patient 3) were collected at three different time points: (1) 2 weeks to 3 months after dose 2 vaccination, (2) 2 weeks to 3 months after dose 3 vaccination, and (3) > 6 months after dose 3 vaccination. The blood samples of the last patient (patient 5) were collected at two different time points: (1) 2 weeks to 3 months after dose 2 vaccination and (2) 2 weeks to 3 months after dose 3 vaccination.

### Antibody response after vaccination with inactivated SARS-CoV-2

**Figure 1:**
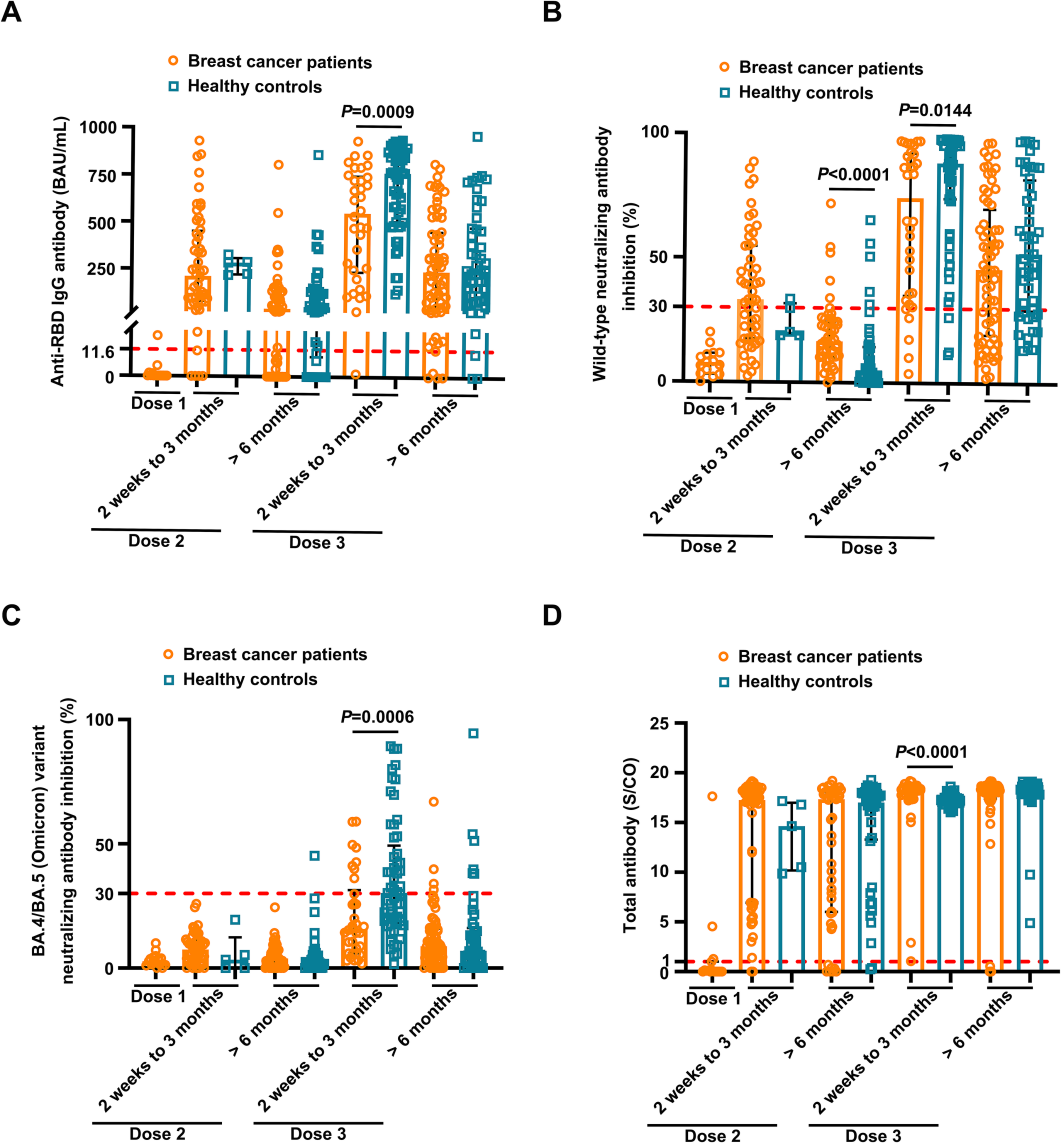
Comparison of antibody responses to SARS-CoV-2 vaccination between breast cancer patients and healthy controls. Comparison of levels of anti-RBD IgG **(A)** and inhibition rates of neutralizing antibodies (NAbs) against both the SARS CoV-2 wild-type virus **(B)** and the BA.4/BA.5 (Omicron) variant **(C)** and levels of anti-SARS-CoV-2 total antibodies **(D)** between the breast cancer patients (BC) and healthy controls (HC) after the first vaccination (BC, *n* = 15), 2 weeks to 3 months after the second vaccination (BC, *n* = 52; HC, *n* = 5), > 6 months after the second vaccination (BC, *n* = 49; HC, *n* = 45), 2 weeks to 3 months after the third vaccination (BC, *n* = 34; HC, *n* = 57), and > 6 months after the third vaccination (BC, *n* = 68; HC, *n* = 48). Bars indicate the median and interquartile range. Statistics were determined using the Mann–Whitney *U*-test.

**Figure 2:**
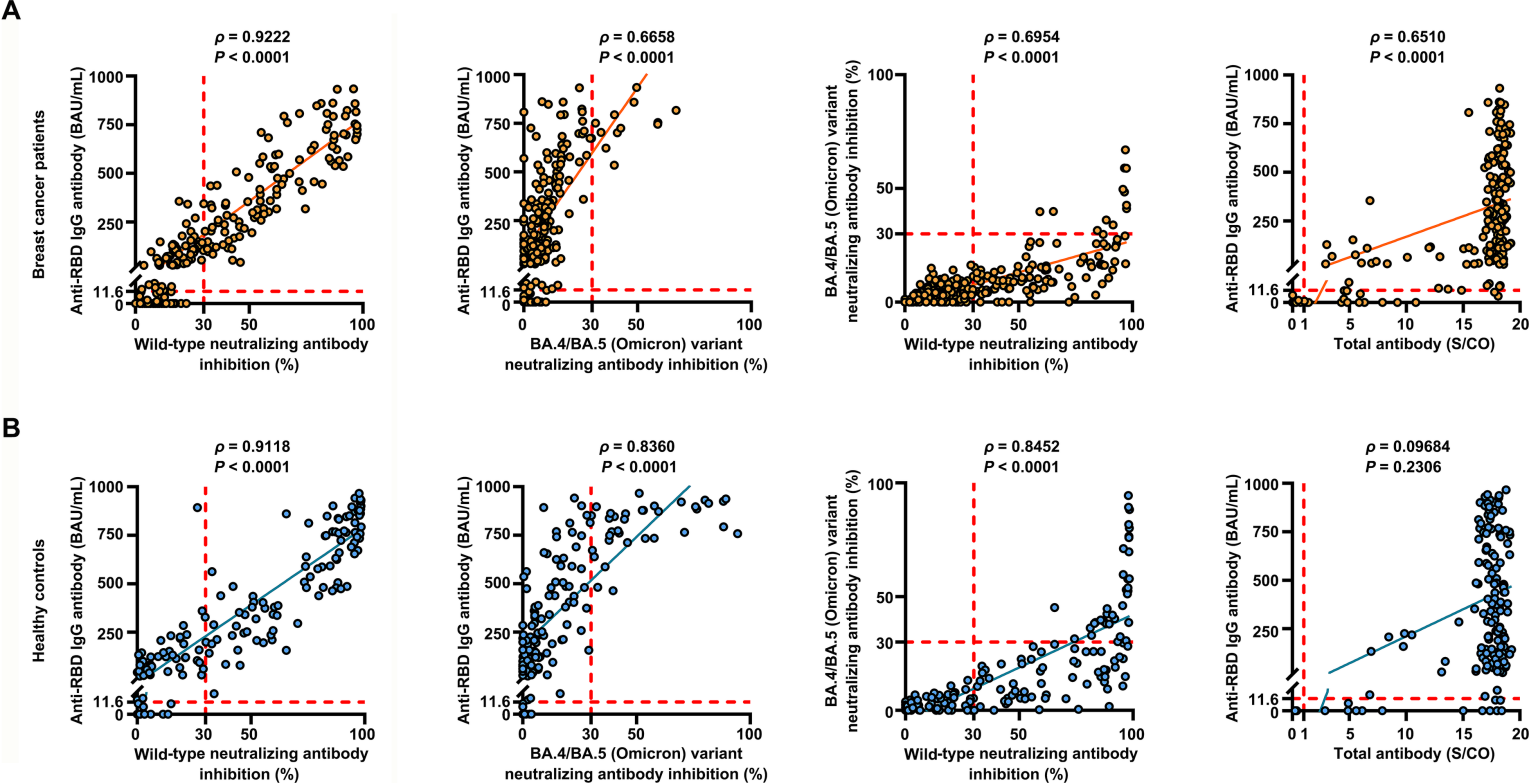
Correlation between the levels of anti-RBD IgG antibodies, inhibition rates of NAbs against both the SARS-CoV-2 wild-type virus and the BA.4/BA.5 (Omicron) variant, and levels of anti-SARS-CoV-2 total antibodies. Correlation between the levels of anti-RBD IgG antibodies, inhibition rates of NAbs against both the SARS-CoV-2 wild-type virus and the BA.4/BA.5 (Omicron) variant, and levels of anti-SARS-CoV-2 total antibodies in breast cancer patients (*n* = 218) **(A)** and healthy controls (*n* = 155) **(B)** determined using Spearman’s rank correlation. Spearman’s correlation coefficient *ρ* provided *p*-values. *p* < 0.05 indicates statistical significance. Each point represents a sample, and the red dashed lines indicate positive detection in the assay.

Overall, the positive anti-SARS-CoV-2 total antibodies, anti-RBD IgG, and NAbs against SARS-CoV-2 BA.4/BA.5 (Omicron) variant responses after both the second and the third vaccinations with inactivated SARS-CoV-2, and positive NAbs against SARS-CoV-2 wild-type virus responses after the third dose were low compared to the healthy controls (Supplementary Table S2). Breast cancer patients had significantly lower anti-RBD IgG antibody titers and NAb inhibition rates against the SARS-CoV-2 wild-type virus and the BA.4/BA.5 (Omicron) variant than healthy controls at 2 weeks to 3 months after dose 3 vaccination (Figures 1A–C). In contrast, total antibody levels at 2 weeks to 3 months after dose 3 vaccination and NAbs against the SARS-CoV-2 wild-type virus at >6 months after dose 2 vaccination were higher among breast cancer patients than healthy controls (Figure 1D). Overall, a strong correlation was observed between anti-RBD IgG antibodies and NAb inhibition rates against the SARS-CoV-2 wild-type virus in breast cancer patients (Figure 2A). Moreover, a strong correlation between anti-RBD IgG antibodies and NAb inhibition rates against the SARS-CoV-2 wild-type virus and the BA.4/BA.5 (Omicron) variant was found. A strong correlation also existed between NAb inhibition rates against the SARS-CoV-2 wild-type virus and NAbs against the BA.4/BA.5 (Omicron) variant among healthy controls (Figure 2B). A moderate correlation was observed between anti-RBD IgG antibodies and inhibition rates of NAbs against the BA.4/BA.5 (Omicron) variant, between NAbs against the SARS-CoV-2 wild-type virus and NAbs against the BA.4/BA.5 (Omicron) variant, and between anti-RBD IgG and total antibodies among breast cancer patients (Figure 2A). Anti-RBD IgG was associated with neutralization, although a subset of vaccine-induced anti-RBD IgG lacked detectable neutralizing capacity. The univariate analysis (Supplementary Table S3) revealed that after the booster dose, older age was associated with decreased anti-RBD IgG-positive responses among breast cancer patients and healthy controls. Supplementary Table S4 indicate that after the booster dose, factors related to reduced wild-type and BA.4/BA.5 (Omicron) variant neutralizing antibody-positive responses in breast cancer patients and healthy controls included older age, breast cancer, and > 6 months after dose 3 vaccination. In multivariable analysis (Table 1 and Supplementary Table S4), > 6 months after dose 3 vaccinations remained an independent predictor of lower positive responses of NAbs against the SARS-CoV-2 wild-type virus and the BA.4/BA.5 (Omicron) variant. In addition, the lymphocyte count was significantly higher in breast cancer patients than in healthy controls 2 weeks to 3 months after dose 3 vaccination, without any significant difference in lymphocyte subsets (Supplementary Table S2).

**Table 1:** Univariate and multivariate analyses of wild-type neutralizing antibody responses in breast cancer patients and healthy controls after SARS-CoV-2 booster vaccination.

		Positive responses (inhibition ≥ 30%)			
	No.	Univariable analysis OR	*p* value	Multivariable analysis OR	*p* value
		(95% CI)		(95% CI)	
Age	207	0.961 (0.938–0.986)	**0.002**	0.984 (0.953–1.017)	0.339
Inactivated vaccine type					
CoronaVac	129	1 [Reference]			
BBIBP-CorV	58	0.622 (0.297–1.305)	0.209		
CoronaVac+BBIBP-CorV	6	0.434 (0.075–2.513)	0.352		
Missing inactivated vaccine type*	14	-	-		
Study population					
Healthy controls	105	1 [Reference]		1 [Reference]	
Breast cancer patients	102	0.440 (0.219–0.886)	**0.021**	0.676 (0.295–1.551)	0.355
Blood samples					
Drawn 2 weeks to 3 months after 3rd vaccination	91	1 [Reference]		1 [Reference]	
Drawn > 6 months after 3rd vaccination	116	0.223 (0.098–0.510)	**<0.001**	0.303 (0.120–0.760)	**0.01**

- Not available.

* Missing values were not included for statistical analysis.*p* value was considered statistically signifcant in bold text.

### Factors affecting antibody responses in breast cancer patients

**Figure 3:**
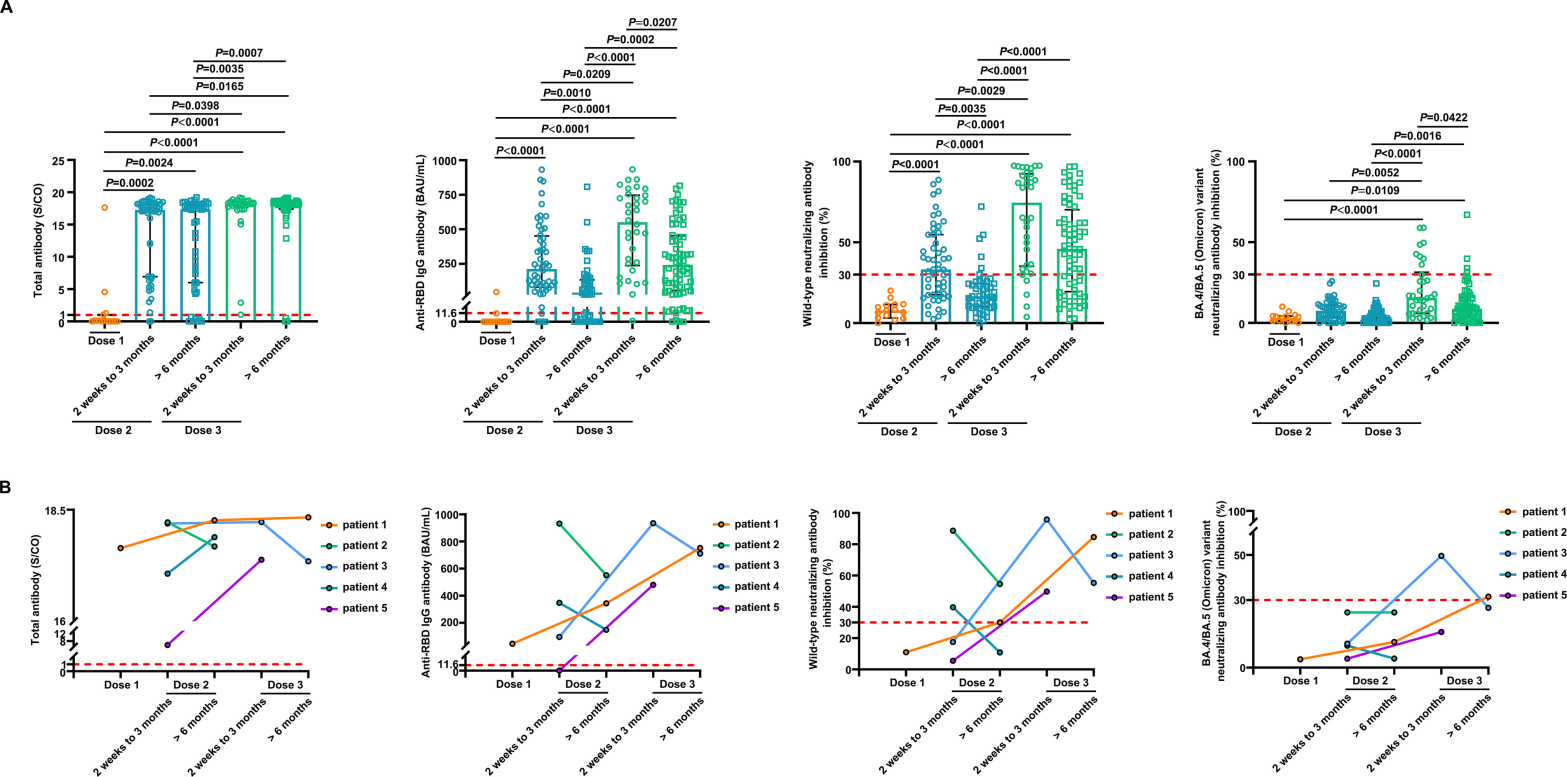
Comparison of antibody responses to SARS-CoV-2 vaccination in patients with breast cancer. **(A)** Comparison of levels of the anti-SARS-CoV-2 total antibodies, anti-RBD IgG, and inhibition rates of NAbs against both the SARS-CoV-2 wild-type virus and the BA.4/BA.5 (Omicron) variant in breast cancer patients after the first vaccination (*n* = 15), 2 weeks to 3 months after the second vaccination (*n* = 52), > 6 months after the second vaccination (*n* = 49), 2 weeks to 3 months after the third vaccination (*n* = 34), and > 6 months after the third vaccination (*n* = 68). Bars indicate the median and interquartile range. Statistics were determined using the Kruskal–Wallis test, followed by Dunn’s multiple comparisons test. **(B)** The anti-SARS-CoV-2 total antibodies, anti-RBD IgG, and inhibition rates of NAbs against both the SARS-CoV-2 wild-type virus and the BA.4/BA.5 (Omicron) variant levels in five breast cancer patients in the longitudinal cohort study. The lines connect the individual samples longitudinally.

**Figure 4:**
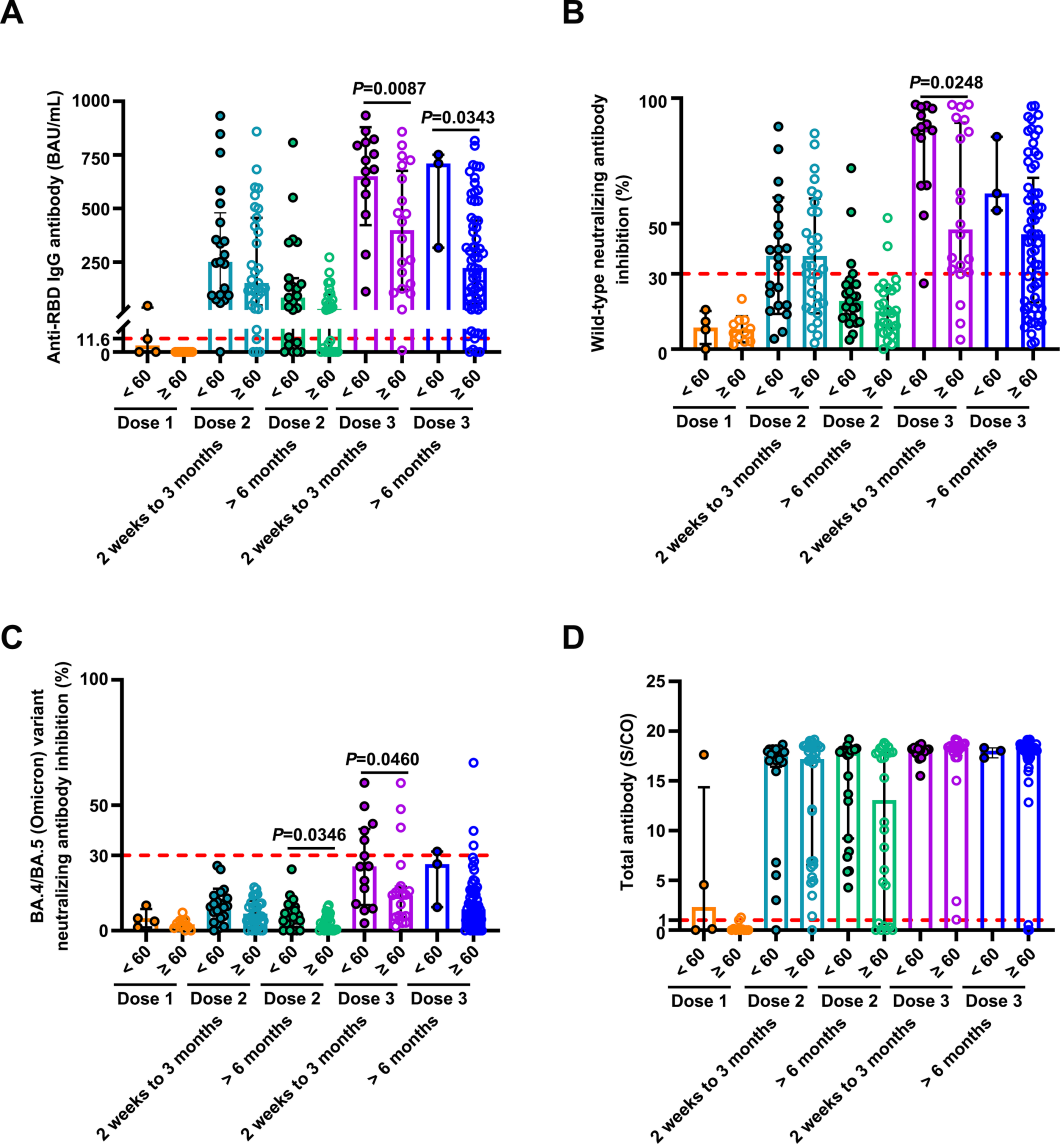
Comparison of antibody responses to SARS-CoV-2 vaccination in patients with breast cancer stratified by age. Comparison of levels of anti-RBD IgG **(A)** and inhibition rates of NAbs against both the SARS-CoV-2 wild-type virus **(B)** and the BA.4/BA.5 (Omicron) variant **(C)**, and levels of anti-SARS-CoV-2 total antibodies **(D)** stratified by age (< 60 and ≥ 60 years) after the first vaccination (< 60 years, *n* = 4, ≥ 60 years, *n* = 11), 2 weeks to 3 months after the second vaccination (< 60 years, *n* = 21; ≥ 60 years, *n* = 31), > 6 months after the second vaccination (< 60 years, *n* = 23; ≥ 60 years, *n* = 26), 2 weeks to 3 months after the third vaccination (< 60 years, *n* = 14; ≥ 60 years, *n* = 20), and > 6 months after the third vaccination (< 60 years, *n* = 3; ≥ 60 years, *n* = 65). Statistics were determined using the unpaired, two-tailed *t*-test or Mann–Whitney *U*-test. *p* < 0.05 indicates statistical significance. Each point represents a sample, and the red dashed lines indicate positive detection in the assay.

We conducted a detailed analysis of the inactivated vaccine type, clinical characteristics, and treatment to induce SARS-CoV-2 antibodies and better understand the determinants of antibody responses after SARS-CoV-2 vaccination in breast cancer patients. Among these patients, positive anti-SARS-CoV-2 total antibody and anti-RBD IgG antibody responses were lower after the first dose (Supplementary Table S2). The responses to NAbs against the SARS-CoV-2 wild-type virus and the BA.4/BA.5 (Omicron) variant were negative after the first dose. Moreover, the positive responses to NAbs against the BA.4/BA.5 (Omicron) variant were negative after the second dose but increased in 24% of patients 2 weeks to 3 months after the third vaccine dose. However, it decreased in 6% of patients at >6 months after the third vaccine (Supplementary Table S2); 2 weeks to 3 months after two vaccine doses, NAbs were detectable against the SARS-CoV-2 wild-type virus in 58% of the patients, whereas they were detectable in 12% of patients >6 months after two vaccine doses. Detectable NAbs against the SARS-CoV-2 wild-type virus improved in 85% of patients at 2 weeks to 3 months following the third vaccine dose, although they decreased in 66% of the patients at >6 months after the third vaccine (Supplementary Table S2). Levels of anti-SARS-CoV-2 total antibodies, anti-RBD IgG, and NAb inhibition rates against the SARS-CoV-2 wild-type virus were significantly lower after the first dose than those at 2 weeks to 3 months after dose 2 (Figure 3A). Anti-RBD IgG and NAbs against the SARS-CoV-2 wild-type virus were significantly decreased at >6 months compared to 2 weeks to 3 months after dose 2 (Figure 3A). Moreover, levels of anti-SARS-CoV-2 total antibodies and anti-RBD IgG and NAb inhibition rates against both the SARS-CoV-2 wild-type virus and the BA.4/BA.5 (Omicron) variant were significantly elevated after a booster dose compared to after the second dose (Figure 3A). Levels of anti-RBD IgG and NAb inhibition rates against the SARS-CoV-2 wild-type virus and the BA.4/BA.5 (Omicron) variant were reduced after >6 months than at 2 weeks to 3 months post-booster vaccination. However, no statistical significance was observed in NAb inhibition rates against the SARS-CoV-2 wild-type virus (Figure 3A). A similar trend was observed in our longitudinal cohort study (Figure 3B). Higher age may also affect immune responses, and patients under 60 years of age had significantly higher anti-RBD IgG levels and NAb inhibition rates against the SARS-CoV-2 wild-type virus and the BA.4/BA.5 (Omicron) variant 2 weeks to 3 months post-booster vaccination than the older group (Figures 4A–C). Vaccination efficacy based on positive responses was analyzed in terms of age, different doses, time since vaccination, inactivated vaccine type, histologic type, TNM stage, histologic grade, molecular subtype, time from cancer diagnosis, undergoing current cancer-directed therapy during blood sample collection, and cancer-directed therapy during vaccination. In breast cancer patients, the inactivated vaccine type was correlated with positive NAb responses against the SARS-CoV-2 wild-type virus as well as with positive responses of anti-SARS-CoV-2 total antibodies and anti-RBD IgG 2 weeks to 3 months or > 6 months after dose 2, respectively (Supplementary Table S5). Additionally, levels of anti-SARS-CoV-2 total antibodies and anti-RBD IgG, as well as NAb inhibition rates against the SARS-CoV-2 wild-type virus and the BA.4/BA.5 (Omicron) variant, were significantly lower in BBIBP-CorV recipients than in CoronaVac recipients (Supplementary Figure S1). Patients who survived breast cancer for >5 years had higher anti-RBD IgG positive responses 2 weeks to 3 months after dose 2 (Supplementary Table S5). Patients receiving endocrine therapy or no treatment during the second vaccination exhibited a significantly higher percentage of anti-SARS-CoV-2 total antibody seropositivity than patients receiving other therapies >6 months after dose 2 (Supplementary Table S5). Univariate analysis (Table 2) revealed that after the second and third doses, BBIBP-CorV was related to decreased odds of having positive NAb responses against the SARS-CoV-2 wild-type virus in breast cancer patients compared to CoronaVac. A time of >6 months after the second vaccination was associated with reduced odds of having positive NAb responses against the SARS-CoV-2 wild-type virus. Moreover, a time of 2 weeks to 3 months after the third vaccination was related to elevated odds of having positive NAb responses against the SARS-CoV-2 wild-type virus than 2 weeks to 3 months after the second vaccination. In the multivariable analysis (Table 2), BBIBP-CorV and a time of >6 months after the second vaccination remained independent predictors of lower positive NAb responses against the SARS-CoV-2 wild-type virus. A time of 2 weeks to 3 months after the third vaccination remained an independent predictor of higher positive NAb responses against SARS-CoV-2 wild-type. Univariate analysis revealed that the factors associated with reduced odds of having positive NAb responses against the SARS-CoV-2 wild-type virus included receiving BBIBP-CorV and receiving both CoronaVac and BBIBP-CorV. It also included a time point of>6 months after the second vaccination, as well as other current therapy (such as chemotherapy, chemotherapy plus radiotherapy, endocrine therapy plus abemaciclib to inhibit CDK4/6, and Chinese medicine), and other therapy during the second vaccination (including chemotherapy, pertuzumab and trastuzumab for HER2-positive, and chemotherapy plus pertuzumab and trastuzumab for HER2-positive) among breast cancer patients receiving two vaccination doses (Supplementary Table S6). Multivariate analysis established that BBIBP-CorV was an independent predictor of lower positive NAb responses against the SARS-CoV-2 wild-type virus after two doses. Univariate analysis indicated that a time of >6 months after the third vaccination was correlated with decreased odds of having positive NAb responses against the SARS-CoV-2 wild-type virus among breast cancer patients who received booster doses (Supplementary Table S7). Multivariate analysis did not identify any negative interactions. Univariate analysis indicated that the factors associated with reduced odds of positive NAb responses against the BA.4/BA.5 (Omicron) variant included older age, age ≥ 60 years, and a time of >6 months after the third vaccination (Supplementary Table S8). The multivariate analysis did not confirm any negative interactions.

**Table 2:** Univariate and multivariate analyses of the factors potentially associated with wild-type neutralizing antibody responses in breast cancer patients.

		Positive responses (inhibition ≥ 30%)			
	No.	Univariable analysis OR	*p* value	Multivariable analysis OR	*p* value
		(95% CI)		(95% CI)	
Age	203	1.008 (0.983–1.033)	0.539		
Age < 60 years					
Yes	61	1 [Reference]			
No	142	1.104 (0.605–2.016)	0.746		
Inactivated vaccine type					
CoronaVac	124	1 [Reference]		1 [Reference]	
BBIBP-CorV	72	0.421 (0.233–0.763)	**0.004**	0.358 (0.148–0.864)	**0.022**
CoronaVac/BBIBP-CorV	7	0.236 (0.044–1.265)	0.092	0.181 (0.012–2.799)	0.221
Blood samples					
Drawn 2 weeks to 3 months after 2nd vaccination	52	1 [Reference]		1 [Reference]	
Drawn > 6 months after 2nd vaccination	49	0.102 (0.037–0.283)	**<0.001**	0.132 (0.040–0.438)	**0.001**
Drawn 2 weeks to 3 months after 3rd vaccination	34	4.253 (1.420–12.739)	**0.010**	5.484 (1.508-19.944)	**0.010**
Drawn > 6 months after 3rd vaccination	68	1.435 (0.681–3.022)	0.342	2.804 (0.968–8.116)	0.057
Histologic type					
Carcinoma in situ	25	1 [Reference]			
Invasive ductal carcinoma	135	0.953 (0.404–2.251)	0.913		
Others	10	1.310 (0.255–6.715)	0.746		
Missing data*	33	-	-		
TNM staging					
0-II	120	1 [Reference]			
III–IV	33	0.961 (0.445–2.078)	0.920		
Missing data*	50				
Histologic grade					
G1	15	1 [Reference]			
G2	79	0.853 (0.282–2.579)	0.778		
G3	45	1.000 (0.310–3.226)	1.000		
Missing data*	64	-	-		
Molecular subtype					
Luminal A	36	1 [Reference]		1 [Reference]	
Luminal B	73	1.895 (0.843–4.259)	0.122	1.267 (0.454–3.534)	0.651
HER2 over-expression subtype/Triple negative	29	2.671 (0.955–7.476)	0.061	1.206 (0.343–4.235)	0.770
Missing data*	65	-	-		
Time from cancer diagnosis to study recruitment, years					
≤ 5	115	1 [Reference]			
> 5	88	0.946 (0.542–1.652)	0.846		
Current cancer-directed therapy					
None	29	1 [Reference]			
Endocrine therapy	126	0.598 (0.258–1.387)	0.231		
Other therapy#	8	0.316 (0.062–1.601)	0.164		
Missing data*	40	-	-		

- Not available.

* Missing values were not included for statistical analysis.

# Chemotherapy, Chemotherapy + Radiotherapy, Endocrine therapy+Abemaciclib for inhibiting CDK4/6, Chinese medicine, Endocrine therapy+Chemotherapy + Trastuzumab, and Endocrine therapy + Chemotherapy+Pertuzumab and trastuzumab for HER2-positive.*p* value was considered statistically signifcant in bold text.

### Factors affecting antibody responses in healthy controls

We conducted a detailed analysis of age, inactivated vaccine type, different vaccination doses, and time since vaccination by inducing SARS-CoV-2 antibodies to understand better the determinants of antibody responses after SARS-CoV-2 vaccination among healthy controls. Positive NAb responses against both the SARS-CoV-2 wild-type virus and the BA.4/BA.5 (Omicron) variant were low after the second dose (Supplementary Table S2). NAbs against the SARS-CoV-2 wild-type virus were detected in 95% of individuals at 2 weeks to 3 months after the third vaccine dose. However, this decreased to 75% of patients >6 months after the third vaccination (Supplementary Table S2). NAbs against the BA.4/BA.5 (Omicron) variant were detected in 51% of individuals 2 weeks to 3 months following the third vaccine dose. In contrast, it decreased to 13% of the patients >6 months after the third vaccination (Supplementary Table S2). NAb inhibition rates against the SARS-CoV-2 wild-type virus and the BA.4/BA.5 (Omicron) variant were significantly lower at 2 weeks to 3 months after the third vaccination in the BBIBP-CorV recipients than in the CoronaVac recipients (Supplementary Figure S1). Levels of anti-SARS-CoV-2 total antibodies, anti-RBD IgG, and NAb inhibition rates against the SARS-CoV-2 wild-type virus and the BA.4/BA.5 (Omicron) variant were significantly lower after the second dose than after the third (Supplementary Figure S2). Levels of anti-RBD IgG, and NAb inhibition rates against the wild-type SARS-CoV-2 virus and the BA.4/BA.5 (Omicron) variant were significantly lower at >6 months than 2 weeks to 3 months after the third vaccination (Supplementary Figures S2B–D). Univariate analysis revealed that times at 2 weeks to 3 months and > 6 months after the third vaccination were related to elevated odds of having positive NAb responses against the BA.4/BA.5 (Omicron) variant than at 2 weeks to 3 months post-second vaccination in the healthy controls (Supplementary Table S9).

## Discussion

This study found that breast cancer patients who received an inactivated vaccine booster dose exhibited higher positive anti-SARS-CoV-2 total antibodies and anti-RBD IgG antibody responses, and positive NAb responses against both the SARS CoV-2 wild-type virus and the BA.4/BA.5 (Omicron) variant responses than those who received only two inactivated vaccines. However, the effect was blunted compared to healthy controls. In addition, older age, breast cancer, and a time of >6 months after the third dose were significantly correlated with undetectable NAbs against the SARS-CoV-2 wild-type virus and the BA.4/BA.5 (Omicron) variant after the third dose in breast cancer patients and healthy controls. Although the SARS-CoV-2 Omicron variant can escape vaccine-induced immunity, our analyses present important evidence that inactivated vaccine boosters are associated with a significant improvement in NAb inhibition rates against the SARS-CoV-2 BA.4/BA.5 (Omicron) variant in breast cancer patients, indicating improvements in protection against the Omicron variant compared to one or two vaccine doses. This finding is important for breast cancer patients who could have impaired vaccine-induced immunity.

Accumulating evidence concerning antibody kinetics post-vaccination indicates that antibodies increase significantly at day 14 and peak at 28 days. This is followed by a decrease at a consistent (IgG antibodies) and rapid (NAbs) rate for the first 3 months, substantially decreasing over 6 months (12, 13). The multivariate analysis revealed that 2 weeks to 3 months after the third dose was an independent predictor of detectable NAbs against the SARS-CoV-2 wild-type virus compared to after the second dose. In contrast, > 6 months after the second dose was an independent predictor of undetectable NAbs against the SARS-CoV-2 wild-type virus. Additionally, the univariate analysis indicated that >6 months after vaccination was correlated with undetectable NAbs against the SARS-CoV-2 wild-type virus compared to 2 weeks to 3 months in patients who received two or three doses, respectively. Furthermore, the univariate analysis demonstrated that >6 months after being vaccinated was associated with undetectable NAbs against the BA.4/BA.5 (Omicron) variant compared to 2 weeks to 3 months in those who received booster doses. Therefore, vaccine efficacy changes over time, with waning immunity.

Previous studies showed that breast and lung cancer patients 60 years or older who received two doses of the CoronaVac vaccine had reduced anti-RBD IgG antibody responses (6). However, we did not observe a statistically significant association between age and antibody-positive responses in breast cancer patients receiving one or two doses. This is likely because our study participants included only breast cancer patients and not lung cancer patients. In contrast, breast cancer patients under 60 years had higher levels of anti-RBD IgG antibodies and NAbs against both wild-type and the BA.4/BA.5 (Omicron) variant at 2 weeks to 3 months after the third dose compared to the older group. Furthermore, univariate analysis revealed the positive NAb responses against the BA.4/BA.5 (Omicron) variant in breast cancer patients who received booster doses were age-dependent. Patients aged 60 years or older had undetectable NAbs against the BA.4/BA.5 (Omicron) variant. These data indicate that patients aged 60 years or older are at high risk. The immune responses of breast cancer patients over 60 years were affected after receiving a third SARS-CoV-2 vaccination. Thus, extensive protective measures should be considered for this population.

Several studies have established that breast cancer patients receiving chemotherapy, CDK4/6 inhibitors, and trastuzumab had attenuated immune responses after the second vaccination (5, 9). Our univariate analysis indicated that currently undergoing another therapy during the collection of blood samples (such as chemotherapy, chemotherapy plus radiotherapy, endocrine therapy plus abemaciclib to inhibit CDK4/6, and Chinese medicine), and other treatments during the second vaccination (such as chemotherapy, pertuzumab, and trastuzumab for HER2-positive cancer, and chemotherapy, pertuzumab and trastuzumab for HER2-positive cancer) were related to undetectable NAbs against the SARS-CoV-2 wild-type virus after two doses. We did not observe any association between them after three doses, which could be due to the relatively small size of this cohort. Our data showed that endocrine therapy, either during the collection of blood samples or during vaccination, did not affect the immune responses to vaccination. The frequency of anti-RBD IgG antibody positivity was significantly lower at 2 weeks to 3 months after two doses among patients diagnosed within 5 years than those diagnosed >5 years. Furthermore, histologic type, TNM stage, histologic grade, and molecular subtype did not affect the humoral immune response to two and three doses of the SARS-CoV-2 vaccine. In particular, univariate and multivariate analyses showed that breast cancer patients who received CoronaVac had significantly higher rates of NAbs against SARS CoV-2 wild-type than those who received BBIBP-CorV. Therefore, CoronaVac should be the preferred vaccine for breast cancer patients.

Our analysis had several limitations. This study was mainly a cross-sectional study. Our data primarily indicated an association, but the ability to infer causality and estimate changes in antibody levels across single individuals over time was limited. However, longitudinal blood samples from five patients reflected similar changes in antibody levels. The detection of NAbs in this study was based on a surrogate competitive binding assay involving binding to RBD and ACE2 and was not the gold standard method of neutralizing live viruses. The number of patients who received the BBIBP-CorV vaccine and were undergoing chemotherapy and targeted therapy was small, indicating the risk of selection bias and the need for more patients in future studies. No data were provided on T cell-induced immune responses after vaccination against the SARS-CoV-2 virus, which could play a key role in providing protective immunity, preventing SARS-CoV-2 infection and severe COVID-19.

## Conclusion

A third inactivated vaccine dose significantly enhanced NAbs against the SARS-CoV-2 wild-type virus, especially the Omicron variant, in breast cancer patients. This response was lower than the healthy controls. Therefore, a third inactivated vaccine dose would benefit breast cancer patients due to the current prevalence of Omicron and reflect the prioritization of breast cancer patients for booster dose delivery, which should not be delayed. However, a significant waning of humoral responses was observed >6 months after receiving both the second and third doses of the inactivated vaccine. Vaccination responses could be insufficient in breast cancer patients over 60 years and in those undergoing chemotherapy and targeted therapy. Therefore, additional strategies should be urgently pursued, such as strict measures to prevent the infection, a higher dose of booster vaccines, and the administration of mAbs to protect these patients.

## Data Availability

The original contributions presented in the study are included in the article/Supplementary material, further inquiries can be directed to the corresponding author.

## Supplementary material

SUPPLEMENTARY FIGURE S1 Comparison of antibody responses to two different SARS-CoV-2 inactivated vaccines. **(A)** Comparison of levels of anti-SARS-CoV-2 total antibodies, anti-RBD IgG, and inhibition rates of NAbs against both SARS-CoV-2 wild-type virus and the BA.4/BA.5 (Omicron) variant in breast cancer patients after the first vaccination with CoronaVac or BBIBP-CorV (CoronaVac, *n* = 8; BBIBP-CorV, *n* = 7), 2 weeks to 3 months after the second vaccination (CoronaVac, *n* = 30; BBIBP-CorV, *n* = 22), > 6 months after the second vaccination (CoronaVac, *n* = 29; BBIBP-CorV, *n* = 17), 2 weeks to 3 months after the third vaccination (CoronaVac, *n* = 21; BBIBP-CorV, *n* = 12), and > 6 months after the third vaccination (CoronaVac *n* = 44, BBIBP-CorV *n* = 21). **(B)** Comparison of levels of anti-SARS-CoV-2 total antibodies, anti-RBD IgG, and inhibition rates of NAbs against both SARS-CoV-2 wild-type virus and the BA.4/BA.5 (Omicron) variant in healthy controls > 6 months after the second vaccination with CoronaVac or BBIBP-CorV (CoronaVac, *n* = 33; BBIBP-CorV, *n* = 10), 2 weeks to 3 months after the third vaccination (CoronaVac, *n* = 41; BBIBP-CorV, *n* = 15), and > 6 months after the third vaccination (CoronaVac, *n* = 23; BBIBP-CorV, *n* = 10). Statistics were determined using the unpaired, two-tailed t-test or Mann-Whitney *U* test. *p* < 0.05 indicates statistical significance. Each point represents a sample, and the red dashed lines indicate positive detection in the assay.

SUPPLEMENTARY FIGURE S2 Comparison of antibody responses to SARS-CoV-2 vaccination in healthy controls. Comparison of levels of anti-SARS-CoV-2 total antibodies **(A)** and anti-RBD IgG **(B)**, and inhibition rates of NAbs against both SARS-CoV-2 wild-type virus **(C)** and the BA.4/BA.5 (Omicron) variant **(D)** in healthy controls 2 weeks to 3 months after the second vaccination (*n* = 5), > 6 months after the second vaccination (*n* = 45), 2 weeks to 3 months after the third vaccination (*n* = 57), and > 6 months after the third vaccination (*n* = 48). Bars indicate the median and interquartile range. Statistics were determined using the Kruskal-Wallis test, followed by Dunn’s multiple comparisons test. *p* < 0.05 indicates statistical significance. Each point represents a sample, and the red dashed lines indicate positive detection in the assay.

SUPPLEMENTARY FIGURE S3 Gating strategies for lymphocytes. Representative flow cytometry plots of lymphocyte subsets.
