# Supplementary figures and images for: COVID-19 inactivated booster vaccines elicit strong protection against SARS-CoV-2 wild-type and Omicron variant in patients with breast cancer
Source: Front Med (Lausanne). 2025 Apr 1;12:1516492. doi: 10.3389/fmed.2025.1516492 (PMC11996645; doi:10.3389/fmed.2025.1516492)

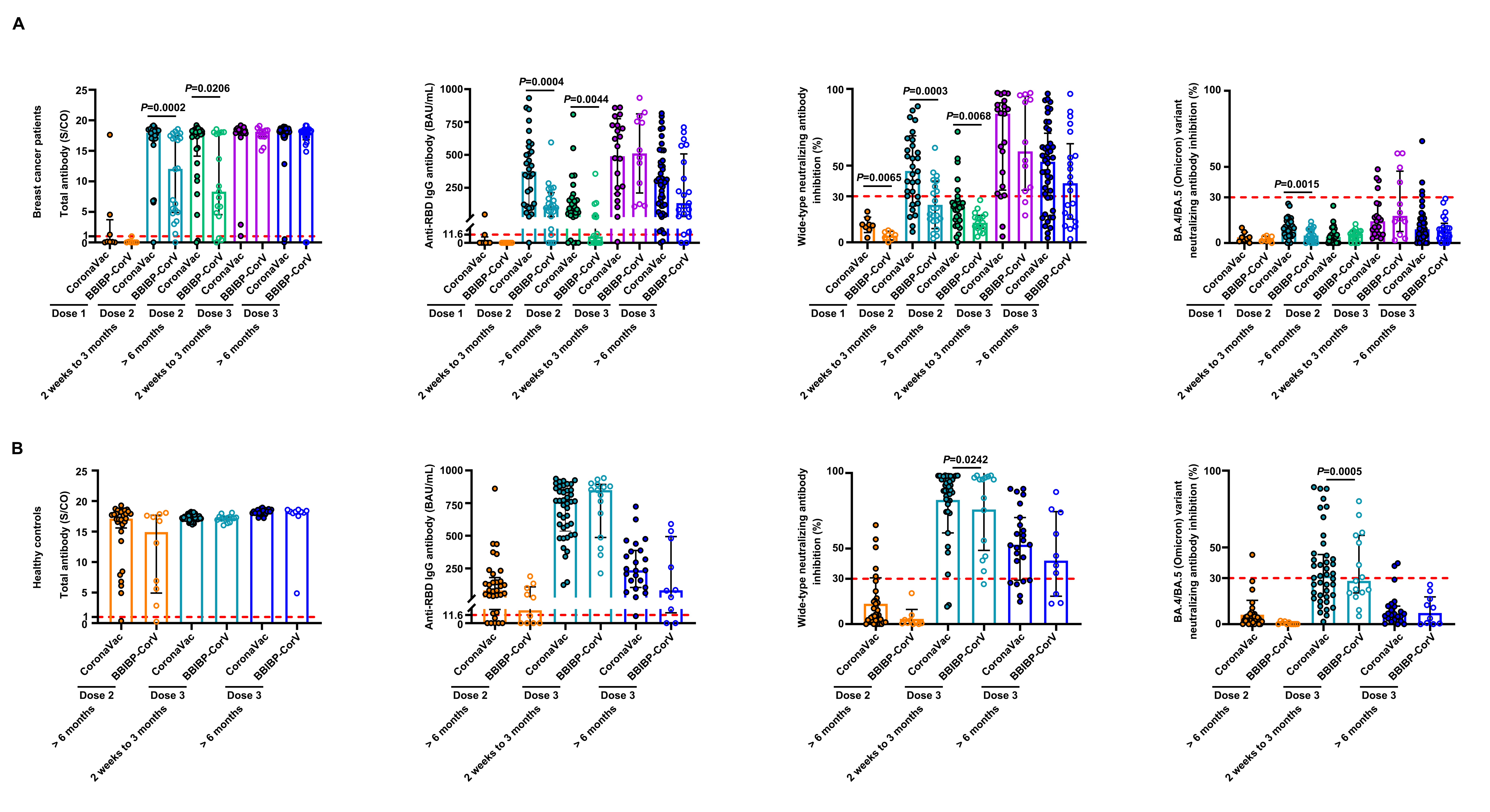

Supplement: SUPPLEMENTARY FIGURE S1 — Comparison of antibody responses to two different SARS-CoV-2 inactivated vaccines. (A) Comparison of levels of anti-SARS-CoV-2 total antibodies, anti-RBD IgG, and inhibition rates of NAbs against both SARS-CoV-2 wild-type virus and the BA.4/BA.5 (Omicron) variant in breast cancer patients after the first vaccination with CoronaVac or BBIBP-CorV (CoronaVac, n = 8; BBIBP-CorV, n = 7), 2 weeks to 3 months after the second vaccination (CoronaVac, n = 30; BBIBP-CorV, n = 22), > 6 months after the second vaccination (CoronaVac, n = 29; BBIBP-CorV, n = 17), 2 weeks to 3 months after the third vaccination (CoronaVac, n = 21; BBIBP-CorV, n = 12), and > 6 months after the third vaccination (CoronaVac n = 44, BBIBP-CorV n = 21). (B) Comparison of levels of anti-SARS-CoV-2 total antibodies, anti-RBD IgG, and inhibition rates of NAbs against both SARS-CoV-2 wild-type virus and the BA.4/BA.5 (Omicron) variant in healthy controls > 6 months after the second vaccination with CoronaVac or BBIBP-CorV (CoronaVac, n = 33; BBIBP-CorV, n = 10), 2 weeks to 3 months after the third vaccination (CoronaVac, n = 41; BBIBP-CorV, n = 15), and > 6 months after the third vaccination (CoronaVac, n = 23; BBIBP-CorV, n = 10). Statistics were determined using the unpaired, two-tailed t-test or Mann-Whitney U test. p < 0.05 indicates statistical significance. Each point represents a sample, and the red dashed lines indicate positive detection in the assay. [file Image_1.TIF]

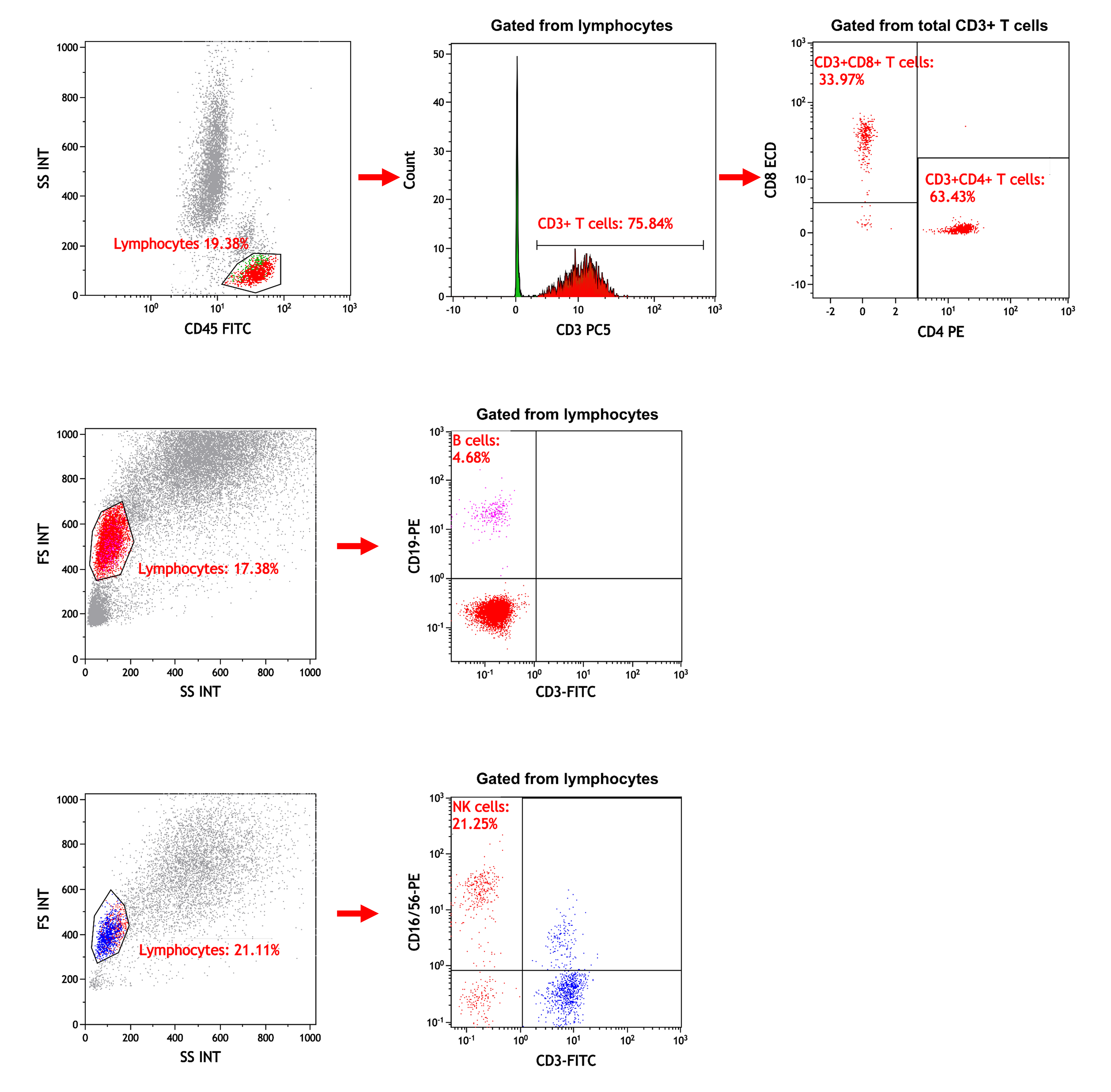

Supplement: SUPPLEMENTARY FIGURE S3 — Gating strategies for lymphocytes. Representative flow cytometry plots of lymphocyte subsets. [file Image_3.TIF]
