# Supplementary material for: COVID-19 inactivated booster vaccines elicit strong protection against SARS-CoV-2 wild-type and Omicron variant in patients with breast cancer
Source: Front Med (Lausanne). 2025 Apr 1;12:1516492. doi: 10.3389/fmed.2025.1516492 (PMC11996645; doi:10.3389/fmed.2025.1516492)
Supplement: Supplementary file 4 [file Table_1.DOCX]

**Table S1. Clinical characteristics of breast cancer patients and healthy controls**

|  | **No. (%)** | |  |
| --- | --- | --- | --- |
|  | **Patients with breast cancer** | **Healthy controls** | ***P*-value** |
|  | **(*n* = 211)** | **(*n* = 155)** |  |
| **Median age, years [IQR]** | 64 [56-69] | 37 [30-53] | **<0.0001^a^** |
| **Sex** |  |  |  |
| Female | 211 (100) | 155 (100) |  |
| **Inactivated vaccine type** | 211 (100) | 155 (100) | 0.2415^b^ |
| CoronaVac | 128 (61) | 99 (64) |  |
| BBIBP-CorV | 76 (36） | 37 (24) |  |
| CoronaVac+BBIBP-CorV | 7 (3) | 2 (1) |  |
| Missing inactivated vaccine type* | 0 | 17 (11) |  |
| **Histologic type** |  |  |  |
| Carcinoma in situ | 26 (12) | NA |  |
| Invasive ductal carcinoma | 139 (66) | NA |  |
| Others | 11 (5) | NA |  |
| Missing data | 35 (17) | NA |  |
| **TNM staging** |  |  |  |
| 0 | 22 (10) | NA |  |
| I | 39 (19) | NA |  |
| II | 59 (28) | NA |  |
| III | 25 (12) | NA |  |
| IV | 11 (5) | NA |  |
| Missing data | 55 (26) | NA |  |
| **Histologic grade** |  |  |  |
| G1 | 16 (8) | NA |  |
| G2 | 82 (39) | NA |  |
| G3 | 46 (22) | NA |  |
| Missing data | 67 (31) | NA |  |
| **Molecular subtype** |  |  |  |
| Luminal A | 37 (18) | NA |  |
| Luminal B | 77 (36) | NA |  |
| HER2 over-expression subtype | 12 (6) | NA |  |
| Triple negative | 17 (8) | NA |  |
| Missing data | 68 (32) | NA |  |
| **Time from cancer diagnosis to study recruitment, years** |  |  |  |
| ≤ 5 | 119 (56) | NA |  |
| > 5 | 92 (44) | NA |  |
| **Blood samples** | 218 | 155 |  |
| Drawn after 1st vaccination | 15 (7) | 0 |  |
| Drawn 2 weeks to 3 months after 2nd vaccination | 52 (24) | 5 (3) |  |
| Drawn > 6 months after 2nd vaccination | 49 (22) | 45 (29) |  |
| Drawn 2 weeks to 3 months after 3rd vaccination | 34 (16) | 57 (37) |  |
| Drawn > 6 months after 3rd vaccination | 68 (31) | 48 (31) |  |
| Drawn after 1st vaccination, median age, years [IQR] | 69 [50-74] | 0 |  |
| Drawn 2 weeks to 3 months after 2nd vaccination, mean age, years [SD] | 58.98 [12.53] | 32.40 [2.302] | **<0.0001^c^** |
| Drawn > 6 months after 2nd vaccination, median age, years [IQR] | 60.0 [49.0-66.5] | 33.0 [29.0-37.5] | **<0.0001^a^** |
| Drawn 2 weeks to 3 months after 3rd vaccination, median age, years [IQR] | 61.0 [47.75-68.25] | 34.0 [27.5-39.5] | **<0.0001^a^** |
| Drawn > 6 months after 3rd vaccination, median age, years [IQR] | 66.0 [62.25-70.0] | 62.0 [41.25-66.0] | **0.0002^a^** |
| Drawn after 1st vaccination, mean days [SD] | 25.20 [12.27] | 0 |  |
| Drawn 2 weeks to 3 months after 2nd vaccination, mean days [SD] | 60.58 [23.37] | 63.20 [30.51] | 0.8160^d^ |
| Drawn > 6 months after 2nd vaccination, median days [IQR] | 251.0 [210.5-330.5] | 280.0 [221.0-328.0] | 0.3930^a^ |
| Drawn 2 weeks to 3 months after 3rd vaccination, median days [IQR] | 65.0 [39.0-80.5] | 74.0 [52.0-90.0] | 0.3057^a^ |
| Drawn > 6 months after 3rd vaccination, median days [IQR] | 229.5 [202.0-260.8] | 243.0 [218.5-265.8] | 0.2521^a^ |
| **Cancer-directed therapy at 1st vaccination among patients drawn blood samples after 1st vaccination** | 15 | NA |  |
| Endocrine therapy | 11 (73) | NA |  |
| Chemotherapy | 1 (7) | NA |  |
| Missing data | 3 (20) | NA |  |
| **Cancer-directed therapy at 2nd vaccination among patients drawn blood samples after 2nd vaccination** | 101 | NA |  |
| Endocrine therapy | 68 (67) | NA |  |
| Chemotherapy | 1 (1) | NA |  |
| Pertuzumab and trastuzumab for HER2-positive | 1 (1) | NA |  |
| Chemotherapy+Pertuzumab and trastuzumab for HER2-positive | 1 (1) | NA |  |
| None | 14 (14) | NA |  |
| Missing data | 16 (16) | NA |  |
| **Cancer-directed therapy at 3rd vaccination among patients drawn blood samples after 3rd vaccination** | 102 | NA |  |
| Endocrine therapy | 60 (59) | NA |  |
| Chemotherapy | 1 (1) | NA |  |
| None | 15 (15) | NA |  |
| Missing data | 26 (25) | NA |  |
| **Current cancer-directed therapy (at the time of plasma collection)** | 218 | NA |  |
| Endocrine therapy | 137 (63) | NA |  |
| Chemotherapy | 3 (1) | NA |  |
| Chemotherapy + Radiotherapy | 1 (0.5) | NA |  |
| Endocrine therapy+Abemaciclib for inhibiting CDK4/6 | 1 (0.5) | NA |  |
| Endocrine therapy+Chemotherapy + Trastuzumab | 1 (0.5) | NA |  |
| Endocrine therapy + Chemotherapy+Pertuzumab and trastuzumab for HER2-positive | 1 (0.5) | NA |  |
| Chinese medicine | 2 (1) | NA |  |
| None | 29 (13) | NA |  |
| Missing data | 43 (20) | NA |  |

- Not available

a Fisher's exact test.

b Chi-square

c Unpaired *t* test.

d Mann Whitney *U* test.

* Missing values were not included for statistical analysis.

*P* < 0.05 was considered statistically significant in bold text.
