# Supplementary material for: COVID-19 inactivated booster vaccines elicit strong protection against SARS-CoV-2 wild-type and Omicron variant in patients with breast cancer
Source: Front Med (Lausanne). 2025 Apr 1;12:1516492. doi: 10.3389/fmed.2025.1516492 (PMC11996645; doi:10.3389/fmed.2025.1516492)
Supplement: Supplementary file 5 [file Table_2.docx]

**Table S2. Overall study population and available number of samples to assess each study outcome**

|  | **No. (%)** | |  |
| --- | --- | --- | --- |
| **Blood samples** | **Patients with breast cancer** | **Healthy controls** | ***P*-value** |
|  | ***n*=218** | ***n*=155** |  |
| **Study outcome** |  |  |  |
| **Total antibody against SARS-CoV-2 response,**  **OD ≥ 0.19 defined as positivity** |  |  |  |
| blood sample drawn after 1st vaccination | 3/15 (20) | 0 | - |
| blood sample drawn 2 weeks to 3 months after 2nd vaccination | 50/52 (96) | 5/5 (100) | >0.9999^a^ |
| blood sample drawn > 6 months after 2nd vaccination | 42/49 (86) | 43/45 (96) | 0.1619^a^ |
| blood sample drawn 2 weeks to 3 months after 3rd vaccination | 34/34 (100) | 57/57 (100) | >0.9999^a^ |
| blood sample drawn > 6 months after 3rd vaccination | 64/68 (94) | 48/48 (100) | 0.1409^a^ |
| **Anti-RBD IgG response, ≥ 11.6 BAU/ml defined as positivity** |  |  |  |
| blood sample drawn after 1st vaccination | 3/15 (20) | 0 | - |
| blood sample drawn 2 weeks to 3 months after 2nd vaccination | 47/52 (90) | 5/5 (100) | >0.9999^a^ |
| blood sample drawn > 6 months after 2nd vaccination | 32/49 (65) | 33/45 (73) | 0.5036^a^ |
| blood sample drawn 2 weeks to 3 months after 3rd vaccination | 33/34 (97) | 56/56 (100) | 0.3778^a^ |
| blood sample drawn > 6 months after 3rd vaccination | 64/68 (94) | 45/48 (94) | >0.9999^a^ |
| **Neutralizing antibody against SARS-CoV-2 wild-type response,**  **inhibition rates ≥ 30% defined as positivity** |  |  |  |
| blood sample drawn after 1st vaccination | 0/15 (0) | 0 | - |
| blood sample drawn 2 weeks to 3 months after 2nd vaccination | 30/52 (58) | 1/5 (20) | 0.1670^a^ |
| blood sample drawn > 6 months after 2nd vaccination | 6/49 (12) | 5/45 (11) | >0.9999^a^ |
| blood sample drawn 2 weeks to 3 months after 3rd vaccination | 29/34 (85) | 54/57 (95) | 0.2824^a^ |
| blood sample drawn > 6 months after 3rd vaccination | 45/68 (66) | 36/48 (75） | 0.4117^a^ |
| **Neutralizing antibody against SARS-CoV-2 BA.4/BA.5 (Omicron) variant response, inhibition rates ≥ 30% defined as positivity** |  |  |  |
| blood sample drawn after 1st vaccination | 0/15 (0) | 0 | - |
| blood sample drawn 2 weeks to 3 months after 2nd vaccination | 0/52 (0) | 0/5 (0) | >0.9999^a^ |
| blood sample drawn > 6 months after 2nd vaccination | 0/49 (0) | 1/45 (2) | 0.4787^a^ |
| blood sample drawn 2 weeks to 3 months after 3rd vaccination | 8/34 (24) | 29/57 (51) | **0.0148^a^** |
| blood sample drawn > 6 months after 3rd vaccination | 4/68 (6) | 6/48 (13) | 0.3145^a^ |
| **Lymphocyte count, cells/ul** |  |  |  |
| blood sample drawn after 1st vaccination, mean cells/ul [SD] | 1511 [497.2] (*n*=15) | 0 | - |
| blood sample drawn 2 weeks to 3 months after 2nd vaccination, mean cells/ul [SD] | 2038 [470.9] (*n*=51) | 1974 [234.4] (*n*=5) | 0.7664^b^ |
| blood sample drawn > 6 months after 2nd vaccination, median cells/ul [IQR] | 1890 [1520-2310] (*n*=49) | 1700 [1395-2105] (*n*=45) | 0.1472^c^ |
| blood sample drawn 2 weeks to 3 months after 3rd vaccination, mean cells/ul [SD] | 2219 [737.2] (*n*=34) | 1838 [482.1] (*n*=52) | **0.0048^b^** |
| blood sample drawn > 6 months after 3rd vaccination, median cells/ul [IQR] | 1870 [1410-2418] (*n*=68) | 1750 [1405-2035] (*n*=48) | 0.1125^c^ |
| **CD3^+^ T, cells/ul** |  |  |  |
| blood sample drawn after 1st vaccination, mean cells/ul [SD] | 942.6 [359.9] (*n*=11) | 0 | - |
| blood sample drawn 2 weeks to 3 months after 2nd vaccination, mean cells/ul [SD] | 1250 [357.7] (*n*=37) | 1554 [0] (*n*=1) | - |
| blood sample drawn > 6 months after 2nd vaccination, mean cells/ul [SD] | 1320 [383.4] (*n*=44) | 1019 [269.4] (*n*=3) | 0.1905^b^ |
| blood sample drawn 2 weeks to 3 months after 3rd vaccination, median cells/ul [IQR] | 1305 [1086-1919] (*n*=32) | 1280 [1099-1905] (*n*=4) | 0.9420^c^ |
| blood sample drawn > 6 months after 3rd vaccination, median cells/ul [IQR] | 1203 [958.6-1522] (*n*=66) | 1145 [914.4-1367] (*n*=45) | 0.3129^c^ |
| **CD4^+^ T, cells/ul** |  |  |  |
| blood sample drawn after 1st vaccination, mean cells/ul [SD] | 349.4 [168.7] (*n*=11) | 0 | - |
| blood sample drawn 2 weeks to 3 months after 2nd vaccination, median cells/ul [IQR] | 375.8 [285.2-550.5] (*n*=37) | 523.7 (*n*=1) | - |
| blood sample drawn > 6 months after 2nd vaccination, median cells/ul [IQR] | 342.3 [272.5-419.7] (*n*=44) | 363.7 [341.4-591.5] (*n*=3) | 0.3901^c^ |
| blood sample drawn 2 weeks to 3 months after 3rd vaccination, median cells/ul [IQR] | 499.1 [366.9-639.0] (*n*=32) | 516.7 [344.3-1082] (*n*=4) | 0.7159^c^ |
| blood sample drawn > 6 months after 3rd vaccination, median cells/ul [IQR] | 443.6 [348.6-589.1] (*n*=66) | 420.5 [349.8-562.5] (*n*=45) | 0.6515^c^ |
| **CD8^+^ T, cells/ul** |  |  |  |
| blood sample drawn after 1st vaccination, mean cells/ul [SD] | 238.4 [131.4] (*n*=11) | 0 | - |
| blood sample drawn 2 weeks to 3 months after 2nd vaccination, median cells/ul [IQR] | 235.1 [187.9-366.7] (*n*=37) | 510.5 (*n*=1) | - |
| blood sample drawn > 6 months after 2nd vaccination, median cells/ul [IQR] | 342.3 [272.5-419.7] (*n*=44) | 266.8 [116.0-294.0] (*n*=3) | 0.0968^c^ |
| blood sample drawn 2 weeks to 3 months after 3rd vaccination, median cells/ul [IQR] | 316.9 [223.3-518.5] (*n*=32) | 344.7 [220.8-427.8] (*n*=4) | 0.9034^c^ |
| blood sample drawn > 6 months after 3rd vaccination, median cells/ul [IQR] | 227.6 [151.5-429.2] (*n*=66) | 249.5 [193.2-370.3] (*n*=45) | 0.5841^c^ |
| **CD4/CD8 ratio** |  |  |  |
| blood sample drawn after 1st vaccination, mean [SD] | 1.59 [0.6842] (*n*=11) | 0 | - |
| blood sample drawn 2 weeks to 3 months after 2nd vaccination, mean [SD] | 1.778 [0.7693] (*n*=38) | 1.026 [0] (*n*=1) | - |
| blood sample drawn > 6 months after 2nd vaccination, median [IQR] | 1.203 [0.9691-1.916] (*n*=44) | 2.012 [1.279-3.134] (*n*=3) | 0.1284^c^ |
| blood sample drawn 2 weeks to 3 months after 3rd vaccination, median [IQR] | 1.635 [1.021-2.065] (*n*=32) | 2.027 [0.9569-3.346] (*n*=4) | 0.4803^c^ |
| blood sample drawn > 6 months after 3rd vaccination, median cells/ul [IQR] | 1.726 [1.152-2.575] (*n*=66) | 1.592 [1.156-2.068] (*n*=45) | 0.4070^c^ |
| **CD19^+^ B, cells/ul** |  |  |  |
| blood sample drawn after 1st vaccination, mean [SD] | 157.4 [80.88] (*n*=11) | 0 | - |
| blood sample drawn 2 weeks to 3 months after 2nd vaccination, median cells/ul [IQR] | 231.5 [162.2-319.2] (*n*=37) | 274.8 (*n*=1) | - |
| blood sample drawn > 6 months after 2nd vaccination, median cells/ul [IQR] | 238.2 [176.8-371.0] (*n*=44) | 155.6 [147.7-184.8] (*n*=3) | 0.0968^c^ |
| blood sample drawn 2 weeks to 3 months after 3rd vaccination, median cells/ul [IQR] | 225.7 [183.9-326.1] (*n*=32) | 194.7 [175.8-270.0] (*n*=4) | 0.3931^c^ |
| blood sample drawn > 6 months after 3rd vaccination, median cells/ul [IQR] | 223.8 [149.2-338.3] (*n*=66) | 166.3 [130.6-245.6] (*n*=45) | 0.0629^c^ |
| **CD16^+^56^+^ NK, cells/ul** |  |  |  |
| blood sample drawn after 1st vaccination, mean [SD] | 285.3 [130.7] (*n*=9) | 0 | - |
| blood sample drawn 2 weeks to 3 months after 2nd vaccination, median cells/ul [IQR] | 424.7 [300.2-570.4] (*n*=36) | 247 (*n*=1) | - |
| blood sample drawn > 6 months after 2nd vaccination, median cells/ul [IQR] | 237.4 [198.6-375.1] (*n*=40) | 180.1 [144.3-405.0] (*n*=3) | 0.3958^c^ |
| blood sample drawn 2 weeks to 3 months after 3rd vaccination, mean cells [SD] | 384.7 [183.1] (*n*=31) | 327.8 [171.6] (*n*=4) | 0.5604^b^ |
| blood sample drawn > 6 months after 3rd vaccination, median cells/ul [IQR] | 367.1 [235.4-482.1] (*n*=61) | 261.0 [179.2-462.2] (*n*=42) | 0.0643^b^ |

- Not available

a Fisher's exact test.

b Unpaired *t* test.

c Mann Whitney *U* test.

*P* < 0.05 was considered statistically significant in bold text.
