# Supplementary material for: COVID-19 inactivated booster vaccines elicit strong protection against SARS-CoV-2 wild-type and Omicron variant in patients with breast cancer
Source: Front Med (Lausanne). 2025 Apr 1;12:1516492. doi: 10.3389/fmed.2025.1516492 (PMC11996645; doi:10.3389/fmed.2025.1516492)
Supplement: Supplementary file 6 [file Table_3.DOCX]

**Table S3. Univariate analysis of anti-RBD IgG antibody responses in breast cancer patients and healthy controls after SARS-CoV-2 booster vaccination**

|  |  | **Positive responses (≥ 11.6 BAU/mL）** | |
| --- | --- | --- | --- |
|  | **No.** | **Univariable analysis OR** | ***P* value** |
|  |  | **(95% CI)** |  |
| **Age** | 207 | 0.928 (0.863-0.998) | **0.044** |
| **Inactivated vaccine type** |  |  |  |
| CoronaVac | 129 | 1 [Reference] |  |
| BBIBP-CorV | 58 | 0.432 (0.104-1.791) | 0.247 |
| CoronaVac/BBIBP-CorV | 6 | - | - |
| Missing inactivated vaccine type* | 14 | - | - |
| **Study population** |  |  |  |
| Healthy controls | 105 | 1 [Reference] |  |
| Breast cancer patients | 102 | 0.571 (0.133-2.452) | 0.451 |
| **Blood samples** |  |  |  |
| Drawn 2 weeks to 3 months after 3rd vaccination | 91 | 1 [Reference] |  |
| Drawn > 6 months after 3rd vaccination | 116 | 0.173 (0.021-1.433) | 0.104 |

- Not available

* Missing values were not included for statistical analysis.
