# Supplementary material for: COVID-19 inactivated booster vaccines elicit strong protection against SARS-CoV-2 wild-type and Omicron variant in patients with breast cancer
Source: Front Med (Lausanne). 2025 Apr 1;12:1516492. doi: 10.3389/fmed.2025.1516492 (PMC11996645; doi:10.3389/fmed.2025.1516492)
Supplement: Supplementary file 7 [file Table_4.DOCX]

**Table S4. Univariate and multivariate analyses of BA.4/BA.5 (Omicron) variant neutralizing antibody responses in breast cancer patients and healthy controls after SARS-CoV-2 booster vaccination**

|  |  | **Positive responses (inhibition ≥ 30%）** | | | |
| --- | --- | --- | --- | --- | --- |
|  | **No.** | **Univariable analysis OR** | ***P* value** | **Multivariable analysis OR** | ***P* value** |
|  |  | **(95% CI)** |  | **(95% CI)** |  |
| **Age** | 207 | 0.937 (0.916-0.959) | **<0.001** | 0.960 (0.929-0.992) | **0.013** |
| **Inactivated vaccine type** |  |  |  |  |  |
| CoronaVac | 129 | 1 [Reference] |  |  |  |
| BBIBP-CorV | 58 | 0.709 (0.329-1.530) | 0.381 |  |  |
| CoronaVac/BBIBP-CorV | 6 | - | - |  |  |
| Missing inactivated vaccine type* | 14 | - | - |  |  |
| **Study population** |  |  |  |  |  |
| Healthy controls | 105 | 1 [Reference] |  | 1 [Reference] |  |
| Breast cancer patients | 102 | 0.267 (0.129-0.551) | **<0.001** | 0.731 (0.271-1.977) | 0.538 |
| **Blood samples** |  |  |  |  |  |
| Drawn 2 weeks to 3 months after 3rd vaccination | 91 | 1 [Reference] |  | 1 [Reference] |  |
| Drawn > 6 months after 3rd vaccination | 116 | 0.138 (0.064-0.298) | **<0.001** | 0.273 (0.113-0.660) | **0.004** |

- Not available

* Missing values were not included for statistical analysis.
