# Supplementary material for: COVID-19 inactivated booster vaccines elicit strong protection against SARS-CoV-2 wild-type and Omicron variant in patients with breast cancer
Source: Front Med (Lausanne). 2025 Apr 1;12:1516492. doi: 10.3389/fmed.2025.1516492 (PMC11996645; doi:10.3389/fmed.2025.1516492)
Supplement: Supplementary file 9 [file Table_6.DOCX]

**Table S6. Univariate and multivariate analyses of the factors potentially associated with wild-type neutralizing antibody responses in breast cancer patients after two vaccine doses of SARS-CoV-2**

|  |  | **Positive responses (inhibition ≥ 30%）** | | | |
| --- | --- | --- | --- | --- | --- |
|  | **No.** | **Univariable analysis OR** | ***P* value** | **Multivariable analysis OR** | ***P* value** |
|  |  | **(95% CI)** |  | **(95% CI)** |  |
| **Age** | 101 | 0.966 (0.925-1.009) | 0.122 |  |  |
| **Age < 60 years** |  |  |  |  |  |
| Yes | 44 | 1 [Reference] |  |  |  |
| No | 57 | 0.530 (0.195-1.440) | 0.213 |  |  |
| **Inactivated vaccine type** |  |  |  |  |  |
| CoronaVac | 59 | 1 [Reference] |  | 1 [Reference] |  |
| BBIBP-CorV | 39 | 0.202 (0.069-0.588) | **0.003** | 0.164 (0.043-0.620) | **0.008** |
| CoronaVac/BBIBP-CorV | 3 | 0.057 (0.004-0.721) | **0.027** | - | - |
| **Blood samples** |  |  |  |  |  |
| Drawn 2 weeks to 3 months after 2nd vaccination | 52 | 1 [Reference] |  | 1 [Reference] |  |
| Drawn > 6 months after 2nd vaccination | 49 | 0.200 (0.067-0.598) | **0.004** | 0.364 (0.098-1.358) | 0.132 |
| **Histologic type** |  |  |  |  |  |
| Carcinoma in situ | 15 | 1 [Reference] |  |  |  |
| Invasive ductal carcinoma | 68 | 0.397 (0.082-1.927) | 0.252 |  |  |
| Others | 6 | - | - |  |  |
| Missing data* | 12 | - | - |  |  |
| **TNM staging** |  |  |  |  |  |
| 0-II | 60 | 1 [Reference] |  |  |  |
| III-IV | 22 | 0.940 (0.292-3.033) | 0.918 |  |  |
| Missing data* | 19 | - | - |  |  |
| **Histologic grade** |  |  |  |  |  |
| G1 | 9 | 1 [Reference] |  |  |  |
| G2 | 44 | 0.857 (0.155-4.754) | 0.860 |  |  |
| G3 | 24 | 0.694 (0.115-4.203) | 0.691 |  |  |
| Missing data* | 24 | - | - |  |  |
| **Molecular subtype** |  |  |  |  |  |
| Luminal A | 26 | 1 [Reference] |  |  |  |
| Luminal B | 36 | 1.244 (0.413-3.747) | 0.697 |  |  |
| HER2 over-expression subtype/Triple negative | 14 | 1.333 (0.283-6.279) | 0.716 |  |  |
| Missing data* | 25 | - | - |  |  |
| **Time from cancer diagnosis to study recruitment, years** |  |  |  |  |  |
| ≤ 5 | 68 | 1 [Reference] |  |  |  |
| > 5 | 33 | 1.867 (0.622-5.600) | 0.265 |  |  |
| **Current cancer-directed therapy** |  |  |  |  |  |
| None | 14 | 1 [Reference] |  | 1 [Reference] |  |
| Endocrine therapy | 68 | 0.272 (0.033-2.249) | 0.227 | - | - |
| Other therapy# | 5 | 0.051 (0.003-0.770) | **0.032** | - | - |
| Missing data* | 14 | - | - |  |  |
| **Cancer-directed therapy at 2nd vaccination among patients drawn blood samples after 2nd vaccination** |  |  |  |  |  |
| None | 14 | 1 [Reference] |  | 1 [Reference] |  |
| Endocrine therapy | 68 | 0.353 (0.042-2.960) | 0.337 | - | - |
| Other therapy## | 3 | 0.042 (0.002-0.973) | **0.048** | - | - |
| Missing data* | 16 | - | - |  |  |

- Not available

* Missing values were not included for statistical analysis.

# Chemotherapy, Chemotherapy + Radiotherapy, Endocrine therapy+Abemaciclib for inhibiting CDK4/6, and Chinese medicine.

## Chemotherapy, Pertuzumab and trastuzumab for HER2-positive, and Chemotherapy+Pertuzumab and trastuzumab for HER2-positive
