# Supplementary material for: COVID-19 inactivated booster vaccines elicit strong protection against SARS-CoV-2 wild-type and Omicron variant in patients with breast cancer
Source: Front Med (Lausanne). 2025 Apr 1;12:1516492. doi: 10.3389/fmed.2025.1516492 (PMC11996645; doi:10.3389/fmed.2025.1516492)
Supplement: Supplementary file 10 [file Table_7.DOCX]

**Table S7. Univariate and multivariate analyses of the factors potentially associated with wild-type neutralizing antibody responses in breast cancer patients after booster vaccination of SARS-CoV-2**

|  |  | **Positive responses (inhibition ≥ 30%）** | | | |
| --- | --- | --- | --- | --- | --- |
|  | **No.** | **Univariable analysis OR** | ***P* value** | **Multivariable analysis OR** | ***P* value** |
|  |  | **(95% CI)** |  | **(95% CI)** |  |
| **Age** | 102 | 0.966 (0.920-1.015) | 0.169 |  |  |
| **Age < 60 years** |  |  |  |  |  |
| Yes | 17 | 1 [Reference] |  | 1 [Reference] |  |
| No | 85 | 0.134 (0.017-1.065) | 0.057 | 0.227 (0.026-2.023) | 0.184 |
| **Inactivated vaccine type** |  |  |  |  |  |
| CoronaVac | 65 | 1 [Reference] |  |  |  |
| BBIBP-CorV | 33 | 0.600 (0.238-1.514) | 0.279 |  |  |
| CoronaVac/BBIBP-CorV | 4 | 0.300 (0.039-2.314) | 0.248 |  |  |
| **Blood samples** |  |  |  |  |  |
| Drawn 2 weeks to 3 months after 3rd vaccination | 34 | 1 [Reference] |  | 1 [Reference] |  |
| Drawn > 6 months after 3rd vaccination | 68 | 0.337 (0.115-0.987) | **0.047** | 0.539 (0.170-1.711) | 0.294 |
| **Histologic type** |  |  |  |  |  |
| Carcinoma in situ | 10 | 1 [Reference] |  |  |  |
| Invasive ductal carcinoma | 67 | 1.486 (0.342-6.458) | 0.597 |  |  |
| Others | 4 | - | - |  |  |
| Missing data* | 21 | - | - |  |  |
| **TNM staging** |  |  |  |  |  |
| 0-II | 60 | 1 [Reference] |  |  |  |
| III-IV | 11 | 0.812 (0.189-3.479) | 0.779 |  |  |
| Missing data* | 31 | - | - |  |  |
| **Histologic grade** |  |  |  |  |  |
| G1 | 6 | 1 [Reference] |  |  |  |
| G2 | 35 | 0.578 (0.059-5.631) | 0.637 |  |  |
| G3 | 21 | 0.850 (0.077-9.440) | 0.895 |  |  |
| Missing data* | 40 | - | - |  |  |
| **Molecular subtype** |  |  |  |  |  |
| Luminal A | 10 | 1 [Reference] |  |  |  |
| Luminal B | 37 | 2.857 (0.632-12.922) | 0.173 |  |  |
| HER2 over-expression subtype/Triple negative | 15 | 4.333 (0.614-30.570) | 0.141 |  |  |
| Missing data* | 40 | - | - |  |  |
| **Time from cancer diagnosis to study recruitment, years** |  |  |  |  |  |
| ≤ 5 | 47 | 1 [Reference] |  | 1 [Reference] |  |
| > 5 | 55 | 0.449 (0.180-1.120) | 0.086 | 0.593 (0.229-1.536) | 0.282 |
| **Current cancer-directed therapy** |  |  |  |  |  |
| None | 15 | 1 [Reference] |  |  |  |
| Endocrine therapy | 58 | 1.042 (0.288-3.774) | 0.950 |  |  |
| Other therapy# | 3 | - | - |  |  |
| Missing data* | 26 | - | - |  |  |
| **Cancer-directed therapy at 3rd vaccination among patients drawn blood samples after 3rd vaccination** |  |  |  |  |  |
| None | 15 | 1 [Reference] |  |  |  |
| Endocrine therapy | 60 | 1.091 (0.302-3.943) | 0.894 |  |  |
| Other therapy## | 1 | - | - |  |  |
| Missing data* | 26 | - | - |  |  |

- Not available

* Missing values were not included for statistical analysis.

# Chemotherapy, Endocrine therapy+Chemotherapy + Trastuzumab, and Endocrine therapy + Chemotherapy+Pertuzumab and trastuzumab for HER2-positive.

## Chemotherapy.
