# Supplementary material for: COVID-19 inactivated booster vaccines elicit strong protection against SARS-CoV-2 wild-type and Omicron variant in patients with breast cancer
Source: Front Med (Lausanne). 2025 Apr 1;12:1516492. doi: 10.3389/fmed.2025.1516492 (PMC11996645; doi:10.3389/fmed.2025.1516492)
Supplement: Supplementary file 11 [file Table_8.DOCX]

**Table S8. Univariate and multivariate analyses of the factors potentially associated with BA.4/BA.5 (Omicron) variant neutralizing antibody responses in breast cancer patients after booster vaccination of SARS-CoV-2**

|  |  | **Positive responses (inhibition ≥ 30%）** | | | |
| --- | --- | --- | --- | --- | --- |
|  | **No.** | **Univariable analysis OR** | ***P* value** | **Multivariable analysis OR** | ***P* value** |
|  |  | **(95% CI)** |  | **(95% CI)** |  |
| **Age** | 102 | 0.927 (0.876-0.980) | **0.008** | 0.973 (0.872-1.086) | 0.630 |
| **Age < 60 years** |  |  |  |  |  |
| Yes | 17 | 1 [Reference] |  |  |  |
| No | 85 | 0.139 (0.038-0.509) | **0.003** | 0.416 (0.026-6.607) | 0.416 |
| **Inactivated vaccine type** |  |  |  |  |  |
| CoronaVac | 65 | 1 [Reference] |  |  |  |
| BBIBP-CorV | 33 | 0.983 (0.273-3.537) | 0.979 |  |  |
| CoronaVac/BBIBP-CorV | 4 | - | - |  |  |
| **Blood samples** |  |  |  |  |  |
| Drawn 2 weeks to 3 months after 3rd vaccination | 34 | 1 [Reference] |  | 1 [Reference] |  |
| Drawn > 6 months after 3rd vaccination | 68 | 0.203 (0.056-0.733) | **0.015** | 0.362 (0.081-1.613) | 0.182 |
| **Histologic type** |  |  |  |  |  |
| Carcinoma in situ | 10 | 1 [Reference] |  |  |  |
| Invasive ductal carcinoma | 67 | 1.397 (0.158-12.380) | 0.764 |  |  |
| Others | 4 | - | - |  |  |
| Missing data* | 21 | - | - |  |  |
| **TNM staging** |  |  |  |  |  |
| 0-II | 60 | 1 [Reference] |  |  |  |
| III-IV | 11 | 0.567 (0.064-4.985） | 0.609 |  |  |
| Missing data* | 31 | - | - |  |  |
| **Histologic grade** |  |  |  |  |  |
| G1 | 6 | 1 [Reference] |  |  |  |
| G2 | 35 | 0.645 （0.059-7.014） | 0.719 |  |  |
| G3 | 21 | 1.563 （0.146-16.719) | 0.712 |  |  |
| Missing data* | 40 | - | - |  |  |
| **Molecular subtype** |  |  |  |  |  |
| Luminal A | 10 | 1 [Reference] |  |  |  |
| Luminal B | 37 | 1.406 (0.145-13.623) | 0.769 |  |  |
| HER2 over-expression subtype/Triple negative | 15 | 1.385 (0.108-17.670) | 0.802 |  |  |
| Missing data* | 40 | - | - |  |  |
| **Time from cancer diagnosis to study recruitment, years** |  |  |  |  |  |
| ≤ 5 | 47 | 1 [Reference] |  |  |  |
| > 5 | 55 | 0.382 (0.107-1.362) | 0.138 |  |  |
| **Current cancer-directed therapy** |  |  |  |  |  |
| None | 15 | 1 [Reference] |  |  |  |
| Endocrine therapy | 58 | 1.194 (0.229-6.215) | 0.833 |  |  |
| Other therapy | 3 | - | - |  |  |
| Missing data* | 26 | - | - |  |  |
| **Cancer-directed therapy at 3rd vaccination among patients drawn blood samples after 3rd vaccination** |  |  |  |  |  |
| None | 15 | 1 [Reference] |  |  |  |
| Endocrine therapy | 60 | 1.147 (0.221-5.965) | 0.870 |  |  |
| Other therapy | 1 | - | - |  |  |
| Missing data* | 26 | - | - |  |  |

- Not available

* Missing values were not included for statistical analysis.

# Chemotherapy, Endocrine therapy+Chemotherapy + Trastuzumab, and Endocrine therapy + Chemotherapy+Pertuzumab and trastuzumab for HER2-positive.

## Chemotherapy.
