# Supplementary material for: COVID-19 inactivated booster vaccines elicit strong protection against SARS-CoV-2 wild-type and Omicron variant in patients with breast cancer
Source: Front Med (Lausanne). 2025 Apr 1;12:1516492. doi: 10.3389/fmed.2025.1516492 (PMC11996645; doi:10.3389/fmed.2025.1516492)
Supplement: Supplementary file 12 [file Table_9.DOCX]

**Table S9. Univariate analysis of the factors potentially associated with wild-type neutralizing antibody responses in healthy controls**

|  |  | **Positive responses (inhibition ≥ 30%）** | |
| --- | --- | --- | --- |
|  | **No.** | **Univariable analysis OR** | ***P* value** |
|  |  | **(95% CI)** |  |
| **Age** | 155 | 1.016 (0.993-1.040) | 0.173 |
| **Inactivated vaccine type** |  |  |  |
| CoronaVac | 99 | 1 [Reference] |  |
| BBIBP-CorV | 37 | 0.783 (0.364-1.687) | 0.533 |
| CoronaVac/BBIBP-CorV | 2 | - | - |
| Missing inactivated vaccine type* | 17 | - | - |
| **Blood samples** |  |  |  |
| Drawn 2 weeks to 3 months after 2nd vaccination | 5 | 1 [Reference] |  |
| Drawn > 6 months after 2nd vaccination | 45 | 0.500 (0.046-5.404) | 0.568 |
| Drawn 2 weeks to 3 months after 3rd vaccination | 57 | 72 (6.026-860.299) | **0.001** |
| Drawn > 6 months after 3rd vaccination | 48 | 12 (1.219-118.1) | **0.033** |

- Not available

* Missing values were not included for statistical analysis.
